# Exploring Therapeutic Strategies for Pediatric Cystic Fibrosis: An In-Depth Comparative Review

**DOI:** 10.7759/cureus.71913

**Published:** 2024-10-20

**Authors:** Alisha Lakhani, Ruchira Clementina, Zainab Siddiqua, Shailee Shroff, Sravani Bhavanam, Maitrey Hareshkumar Pandya, Bhoomi Bagadia, Usman Khan, Mishal Mohammed Koyappathodi Machingal, Ankush Kimmatkar, Prachi Chunawala, Harpratap Singh, Hasim Reza, Madhavi Katta

**Affiliations:** 1 Research, Research MD, Vadodara, IND; 2 Medicine, Shantabaa Medical College, Amreli, IND; 3 Medicine, Government Medical College, Nizamabad, Nizamabad, IND; 4 Medicine, Gandhi Medical College, Secunderabad, IND; 5 Internal Medicine, Gujarat Medical Education and Research Society (GMERS) Medical College, Himmatnagar, IND; 6 Medicine, Research MD, Vadodara, IND; 7 Medicine, Davao Medical School Foundation, Davao City, PHL; 8 Medicine, KLE Academy of Higher Education and Research (KAHER) Jawaharlal Nehru Medical College, Belagavi, IND; 9 Medicine, Akhtar Saeed Medical and Dental College, Lahore, PAK; 10 Medicine, Government Medical College, Kozhikode, Kozhikode, IND; 11 Internal Medicine, Capital Medical University, Beijing, CHN; 12 Medicine, Jawaharlal Institute of Postgraduate Medical Education and Research, Puducherry, IND; 13 General Medicine, Subharti Medical College, Meerut, IND; 14 Medicine, Central America Health Sciences University, Belize City, BLZ; 15 Medicine, Maharashtra Institute of Medical Education and Research, Pune, IND

**Keywords:** cf diagnosis, cftr mutation, cystic fibrosis (cf), general pediatrics, pharmacologic management

## Abstract

Cystic fibrosis (CF) is a genetic disorder that profoundly affects the respiratory and digestive systems, particularly in pediatric patients. This comprehensive review aims to conduct a comparative analysis of various treatment modalities employed in the management of CF in children.

We systematically evaluated current literature focusing on pharmacological interventions, airway clearance techniques, nutritional management, and emerging therapies, including gene therapy and personalized medicine. Our analysis highlights the efficacy, safety, and accessibility of these treatments through a comparative lens, examining performance across diverse patient populations. Key comparisons include standard therapies, such as CF transmembrane conductance regulator (CFTR) modulators, versus traditional treatments and the effectiveness of airway clearance techniques in relation to lung function outcomes.

We explore variations in treatment adherence and outcomes based on socioeconomic factors and healthcare systems. The review underscores the importance of individualized care plans tailored to the unique needs of pediatric patients. By synthesizing findings from clinical studies, meta-analyses, and expert guidelines, this review serves as a valuable resource for healthcare providers and researchers. Our goal is to optimize therapeutic strategies for pediatric patients with CF and ultimately improve their clinical outcomes.

## Introduction and background

Cystic fibrosis (CF) is a life-threatening genetic disorder that primarily affects the lungs and digestive system. This condition results from a malfunction in the exocrine glands, leading to the production of thick, sticky mucus. The disorder is caused by mutations in the CF transmembrane conductance regulator (CFTR) gene, which regulates the movement of salt and water in and out of cells. The resulting abnormal CFTR protein causes mucus to build up in organs such as the lungs and pancreas, leading to chronic infections, respiratory failure, and pancreatic insufficiency [1].

CF is inherited in an autosomal recessive manner, meaning both parents must carry a defective CFTR gene for the disorder to be passed on to their child. The disease is most common among populations of European descent, with early childhood being the typical period for diagnosis [2]. The clinical manifestations of CF require lifelong medical care, addressing pulmonary complications, digestive issues, and dysfunction in other organs [3].

Given the complexity and chronic nature of CF, there is a critical need for a comprehensive review of current treatment modalities and emerging therapies. Understanding the effectiveness, safety, and long-term impacts of these treatments is essential for improving the quality of life and survival rates for patients with CF. Advances in genetic research have led to the development of novel CFTR modulators, which aim to correct the underlying genetic mutation rather than just alleviate symptoms [4,5].

In recent years, new treatments and clinical trials have focused on CFTR modulator therapies and alternative antibacterial approaches, such as nitric oxide (NO) delivery, to combat infections from CF-related bacteria like *Pseudomonas aeruginosa* and *Staphylococcus aureus* [6]. These breakthroughs represent a shift toward personalized medicine, targeting the root causes of CF and enhancing patient outcomes. However, it remains necessary to evaluate these new treatments through evidence-based studies to ensure their long-term efficacy and safety [7].

Thus, a detailed review of existing and novel therapies is crucial for guiding treatment decisions and optimizing patient care. This review will explore advancements in CFTR modulator therapies and alternative treatments, emphasizing the importance of evidence-based comparisons to achieve the best outcomes for pediatric CF patients [1,5].

## Review

Historical perspective

CF was first described in 1938 by Dr. Dorothy Andersen, a pathologist who identified the disease in infants with a high mortality rate. Initially, CF was characterized by its devastating impact on young children, leading to severe respiratory and digestive issues.

Since then, advances in medical care - including improved diagnostic techniques, better nutritional support, and the establishment of specialized CF care centers - have significantly improved the prognosis for individuals with CF. Today, many people with CF are living well into adulthood, reflecting the remarkable progress made in treatments and management strategies. Key milestones in CF care are summarized in (Table 1).

**Table 1:** Historical Evolution of Treatment of CF CF - cystic fibrosis; CFTR - cystic fibrosis transmembrane conductance regulator; DNase - deoxyribonuclease; FDA - Food and Drug Administration

Timeline	Treatment Strategies
1930s	1938: Discovery of CF by Dr. Dorothy Andersen. Initial focus on symptomatic treatment for malnutrition and used penicillin and sulfonamides for recurrent respiratory infections [8].
1950s-1960s	Development of chest physiotherapy to clear mucus from the lungs. Improved nutritional guidelines by adding pancreatic enzymes. Antistaphylococcal antibiotics were also added. Establishment of specialized CF care centers. Focus on early diagnosis and comprehensive care improved the mortality rate among CF survivors [9].
1970s-1980s	Introduction of pancreatic enzyme supplements to aid digestion. Further refinement of respiratory therapies. The Discovery of the CFTR gene marked an important milestone, which not only helped us better understand the disease pathology but also paved the way for novel treatment (targeted approach) [10].
1990s	Introduction of high-frequency chest wall oscillation (vest therapy). Improved antibiotic use by inhalation route. Chronic use of antibiotics was increased, suggesting a more aggressive take on infections caused by CF pathology. Use of inhaled tobramycin increased [11].
2000s	Introduction of inhaled DNase to reduce mucus viscosity leading to drastic decreases in pulmonary infections. Progress in lung transplantation techniques [12,13].
2010s	First FDA-approved CFTR modulator, ivacaftor, was introduced in 2012. There were significant changes in 28-day sweat challenge test, which further encouraged more drugs from the same class. In 2015, ivacaftor-lumacaftor combination treatment was introduced, which did wonders for patients who were homozygous for Phe508del CFTR mutation [14,15].
Until present	Development of combination CFTR modulator therapies, like elexacaftor/tezacaftor/ivacaftor, has improved patient care and life expectancy. When used together, elexacaftor, tezacaftor, and ivacaftor greatly improved the processing and movement of CFTR proteins in airway cells from people with either two copies of the F508del mutation or one copy of the F508del mutation along with a minimal function mutation. Tezacaftor was chosen over lumacaftor as the second corrector because it has better side effects and fewer drug interactions [16].

Pathophysiology of CF

CF is an autosomal recessive disease caused by mutations in the CFTR gene, which was discovered in 1989. This gene, located on chromosome 7, regulates the movement of chloride and bicarbonate ions across cell membranes. Mutations in the CFTR gene reduce the number or function of chloride channels, leading to the production of thick mucus that affects multiple organs, particularly the lungs, pancreas, liver, and intestines. Lung damage is the most severe, causing death in 90% of CF patients. CF is considered a multisystem disease, presenting significant treatment challenges due to its widespread effects on exocrine tissues [17-19].

Over 2,000 mutations have been identified in the CFTR gene; however, not all of these variations result in CF. These mutations can be classified into pathogenic, benign, or variants of uncertain significance, each having different clinical impacts. The CFTR gene normally produces a 6.5-kilobase mRNA that encodes a protein consisting of 1,480 amino acids, which forms a chloride channel in the membranes of epithelial cells. This channel is crucial for maintaining fluid balance. The most common mutation, delta F508, is present in approximately 70% of White American patients and accounts for two-thirds of all CF cases globally [18,20,21].

The CFTR protein belongs to the adenosine triphosphate (ATP)-binding cassette (ABC) transporter family and is located on the apical surface of epithelial cells. Its primary function is to regulate the movement of chloride ions (Cl⁻) across the apical membranes, facilitating the flow of water into the luminal space through an osmotic gradient. The opening of this chloride channel is controlled by phosphorylation by protein kinase A and the binding of ATP. In addition to chloride transport, the CFTR protein also facilitates bicarbonate (HCO₃⁻) transport, which helps maintain the pH of epithelial surfaces and mucus. In CF, defective CFTR proteins impair both Cl⁻ and HCO₃⁻ transport, resulting in abnormally thick and sticky mucus with a low pH in the airway lumen [19,21].

Moreover, CFTR modulates the activity of the epithelial sodium channel (ENaC), which is the primary route for sodium absorption. In CF, mutations in CFTR lead to excessive ENaC-mediated sodium absorption, further disrupting ion balance. This increased sodium and water absorption reduces the thickness of the airway surface liquid, which is essential for proper ciliary function. The resultant dehydration of airway surfaces impairs mucociliary clearance, leading to the accumulation of thick, viscous mucus. This environment fosters bacterial colonization and biofilm formation, reducing the effectiveness of antimicrobial defenses. The combination of mucus retention, bacterial infection, and inflammation initiates a vicious cycle of airway blockage, chronic inflammation, and infection, progressively damaging lung tissue and ultimately leading to respiratory failure [20-24].

In the pancreas, CFTR mutations disrupt bicarbonate secretion, causing enzyme concentration and mucus plugging of the small pancreatic ducts. This blockage prevents digestive enzymes from reaching the small intestine, resulting in the malabsorption of fats, proteins, and carbohydrates, which manifests as malnutrition in CF patients [19,25].

Clinical manifestations of CF can appear at birth or develop later in life, with symptoms including chronic coughing, recurrent respiratory infections, diarrhea, and poor weight gain. The most common and severe complications occur in the lungs, characterized by bronchiectasis and persistent infection. Exocrine pancreatic insufficiency, leading to malabsorption, hepato-biliary disease, and male infertility, are also prominent features of the disease [18,22].

In summary, CF is a multisystem disease driven by dysfunctional CFTR protein, resulting in abnormal ion transport, excessive mucus production, and chronic inflammation that progressively damages affected organs, particularly the lungs and pancreas. This combination of factors leads to a cascade of complications that define the CF phenotype, with the respiratory system being the most severely affected [21].

Risk factors of CF 

Modifiable risk factors like maternal education, home stability, and early CF therapy significantly improve lung health outcomes in CF, while non-modifiable factors like CFTR genotype and meconium ileus predict poorer outcomes (Table 2).

**Table 2:** Modifiable and Non-Modifiable Risk Factors BMI - body mass index; CF - cystic fibrosis; CFELD - cystic fibrosis early-onset lung disease; QoL - quality of life; PRS - polygenic risk score

Risk Factors	Impact on CF
Modifiable Risk Factors	
Maternal Education Level	Higher maternal education (at or above community college) is associated with a lower risk of severe CFELD [26].
Stable Two-Parent Home	Children in stable two-parent households during the first three years of life show better health outcomes and improved lung function [26].
Health Insurance	Not receiving Medicaid (indicating better private insurance or higher income) is associated with a lower risk of severe CFELD and better access to care [26].
Daycare Attendance	Children attending daycare for ≥20 hours per week have a higher risk of severe CFELD and more frequent pulmonary exacerbations, possibly due to increased exposure to pathogens [26].
Exposure to Smoking	Smoke exposure is linked to increased CFELD severity, contributing to poorer lung function and greater respiratory complications [26].
Weight-for-Age/Height-for-Age z-Scores	Better z-scores at birth and three years are associated with lower likelihood of severe CFELD, emphasizing the critical role of early nutrition for lung development [26].
Poor Growth (BMI <10th Percentile)	Poor growth is linked to worse lung function and more severe lung damage, as malnutrition impairs lung growth and immune function, exacerbating CF symptoms [27].
Early CF Therapy	Early visits to specialized CF centers and longer durations of ivacaftor therapy correlate with improved lung function and overall health outcomes [26].
Hospitalizations	Frequent hospitalizations (within six months) are linked to worse lung outcomes due to exacerbations and complications, leading to potential lasting lung damage [27].
Infection With Mucoid Pseudomonas aeruginosa	Infections with mucoid Pseudomonas aeruginosa are associated with significantly worse lung outcomes, as they form biofilms resistant to antibiotics, complicating treatment. Early eradication can help preserve lung health [27].
Non-Modifiable Risk Factors	
CFTR Genotype	Patients with F508del or other pancreatic insufficiency-associated variants in both alleles have a higher risk of severe CFELD, indicating that more severe mutations lead to poorer outcomes [26].
PRS	Lower PRS z-scores correlate with an increased risk of severe CFELD, indicating the significant influence of genetic modifiers beyond the CFTR gene on disease severity [26].
Meconium Ileus at Birth	Meconium ileus at birth is associated with worse lung function later in life, suggesting more severe CFTR dysfunction and greater respiratory compromise [27,28].

Current treatment landscape

The current medical management of CF includes a combination of mucolytics, bronchodilators, antibiotics, and CFTR modulators. Since the update of the guidelines in 2013, additional CFTR modulators have received FDA approval, and this area continues to be a significant focus of research and development in the treatment of CF [29].

Since the identification of the CFTR gene in 1989, extensive efforts have been made to develop therapies that address the underlying dysfunctions caused by CFTR mutations, aiming to target the pathogenesis cascade more effectively. Advances in cell-based high-throughput screening have greatly facilitated the rapid identification of CFTR modulators. These medications can enhance or even restore the functional expression of specific CF-causing mutations and are categorized based on their effects on CFTR mutations. The five broad categories include potentiators, which enhance the activity of the CFTR protein at the cell surface; correctors, which assist in the proper folding and trafficking of the CFTR protein to the cell surface; stabilizers, which help maintain the stability of the CFTR protein once at the surface; read-through agents, which enable the CFTR protein to overcome certain types of mutations that create premature stop signals; and amplifiers, which increase the production of CFTR protein [30].

As of now, the four FDA-approved CFTR modulator therapies are ivacaftor, lumacaftor combined with ivacaftor, tezacaftor combined with ivacaftor, and elexacaftor combined with tezacaftor and ivacaftor (Table 3) [29].

**Table 3:** Dosage, Indications, and Contraindications CFTR - cystic fibrosis transmembrane conductance regulator

Medications	Dosage	Mechanism	Indication	Contraindication
Ivacaftor Monotherapy [31,32]	1 to <2 months (≥3 kg): 5.8 mg packet every 12 hours; 2 to <4 months (≥3 kg): 13.4 mg packet every 12 hours; 4 to <6 months (≥5 kg): 25 mg packet every 12 hours; 6 months to <6 years (5 kg to <7 kg): 25 mg packet every 12 hours; 6 months to <6 years (7 kg to <14 kg): 50 mg packet every 12 hours; 6 months to <6 years (≥14 kg): 75 mg packet every 12 hours; 6 years and older: 150 mg tablet every 12 hours	Potentiates CFTR protein channel gating, increasing chloride transport.	Patients with at least one CFTR mutation susceptible to ivacaftor potentiation, regardless of age.	1) Hypersensitivity reactions, including anaphylaxis. 2) Concomitant use with CYP3A inducers.
Lumacaftor + Ivacaftor [33,34]	Age 1 to 2 years: 7 to <9 kg: 75 mg/94 mg every 12 hours 9 to <14 kg: 100 mg/125 mg every 12 hours ≥14 kg: 150 mg/188 mg every 12 hours; age 2 to 5 years: <14 kg: 100 mg/125 mg every 12 hours ≥14 kg: 150 mg/188 mg every 12 hours; age 6 years and older: 6 to 11 years: 2 tablets (100 mg/125 mg) every 12 hours 12 years and older: 2 tablets (200 mg/125 mg) every 12 hours	Increases the conformational stability of the F508del-CFTR protein, raising cell surface expression.	Recommended for patients homozygous for the F508del mutation in the CFTR gene who are at least one year of age.	Absolute contraindications: none. Caution in advanced liver disease, hypersensitivity reactions, and respiratory events.
Tezacaftor + Ivacaftor (SYMDEKO) [35,36]	Age 6 to 11 years, weighing <30 kg: tezacaftor 50 mg/ivacaftor 75 mg each morning; ivacaftor 75 mg each evening; age 6 to 11 years, ≥30 kg and 12 years and older: tezacaftor 100 mg/ivacaftor 150 mg each morning; ivacaftor 150 mg each evening	Improves the amount and functionality of cell surface CFTR.	Recommended for patients 6 years and older who are homozygous for the F508del mutation or have at least one responsive mutation in the CFTR gene.	Hypersensitivity to ivacaftor, tezacaftor, or any components of the formulation.
Elexacaftor + Tezacaftor + Ivacaftor (Trikafta) [37,38]	<30 kg: morning: elexacaftor 50 mg/tezacaftor 25 mg/ivacaftor 37.5 mg; Evening: ivacaftor 75 mg; >30 kg: morning: elexacaftor 100 mg/tezacaftor 50 mg/ivacaftor 75 mg; evening: ivacaftor 150 mg; 12 years and older: morning: elexacaftor 100 mg/tezacaftor 50 mg/ivacaftor 75 mg; evening: ivacaftor 150 mg	Combines one potentiator (ivacaftor) with two correctors (elexacaftor and tezacaftor) to improve CFTR protein amount and function.	Indicated for persons 12 years and older with at least one F508del mutation in the CFTR gene. Provides potential therapy to patients previously excluded from CFTR modulation therapy.	None.

Mucolytics 

Mucolytics play a crucial role in the management of CF by enhancing mucus clearance from the airways. Dornase alfa, marketed as Pulmozyme, is administered as a nebulized treatment at a dosage of 2.5 mg once daily for all age groups, with some individuals requiring it twice daily. This synthetic form of the human enzyme deoxyribonuclease I (DNase) specifically targets and breaks down DNA in mucus, thereby reducing its thickness and facilitating easier clearance from the airways. It is particularly recommended for patients with CF who have a forced vital capacity (FVC) greater than 40% of expected, as it enhances lung function and decreases the incidence of respiratory tract infections requiring parenteral antibiotics. However, patients with a history of anaphylaxis or hypersensitivity to dornase alfa, Chinese hamster ovary cell products, or any components of the product should avoid its use [39].

Nebulized hypertonic saline is another important mucolytic, typically administered as 4 mL of a 7% solution twice daily. For patients who cannot tolerate this concentration, a 3% or 3.5% solution may be used. Nebulized hypertonic saline hydrates mucus, enhances mucociliary clearance, and may alleviate inflammatory processes in the airways. It is indicated for symptomatic and anti-inflammatory relief in conditions like CF, bronchiectasis, and bronchiolitis, which cause mucus thickening and inflammation. However, this treatment should not be given to patients who show intolerance to hypertonic sodium chloride 7% during the initial test dose. Careful monitoring is required for hemoptysis patients and those with significant declines in lung function to ensure safety [40,41].

In summary, both dornase alfa and nebulized hypertonic saline are key mucolytics in CF treatment aimed at improving respiratory function and quality of life for patients.

Antibiotic therapy

Antibiotic therapy is an essential component of managing CF, particularly in pediatric patients. Prophylactic antibiotic treatment is recommended to prevent opportunistic infections from organisms such as Pseudomonas aeruginosa, which can significantly impair lung function and reduce survival rates [29]. For CF patients aged six years and older, if Pseudomonas aeruginosa is consistently identified in airway cultures, preventive antibiotic therapy is advisable. Inhaled aztreonam has been shown to improve lung function and decrease the frequency of pulmonary exacerbations in these patients. Furthermore, azithromycin has demonstrated anti-inflammatory properties, contributing to better clinical outcomes in individuals with CF [42,43].

In summary, the use of prophylactic antibiotics like inhaled aztreonam and azithromycin is vital for maintaining lung health and preventing severe infections in CF patients, ultimately enhancing their quality of life and longevity.

Clinical scoring system 

The clinical scoring system for CF has evolved significantly since the first scoring system was introduced in 1958. Over the years, several scoring systems have been developed, each aiming to assess various clinical parameters. However, many of these systems have not undergone rigorous evaluation and have become outdated [44,45].

Among the existing systems, the Doershuk-modified Shwachman score stands out as one of the most reliable tools for monitoring CF patients. It is particularly effective in characterizing follow-up cases, providing a structured approach to evaluate clinical status and guide treatment decisions. On the other hand, the modified Huang score is more suited for prognostic assessment in patients with end-stage disease, helping clinicians anticipate disease progression and inform end-of-life care options (Table 4) [46-48]. 

**Table 4:** Shwachman-Kulczycki Score [49] cm - centimeters; kg - kilograms

Total Score	Points	General Activity	Physical Examination	Nutrition	X-ray Findings
Excellent (86-100)	25	Normal	Normal	Normal	Normal
Good (71-85)	20	Lack of endurance	Rare cough, no clubbing, minimal hyperinflation	Stools slightly abnormal	Minimal accentuation of bronchovascular markings, early hyperinflation
Mild (56-70)	15	Tires easily after exertion	Occasional cough wheeze, increased respiratory rate early clubbing	Stools often abnormal, minimal abdominal distension, reduced muscle mass	Mild hyperinflation, patchy atelectasis, increased bronchoalveolar markings
Moderate (41-55)	10	Home teacher dyspnea after short walk	Frequent cough, clubbing, moderate hyperinflation crackles and wheezes	weight and height below the 3rd centile, offensive stools, reduced muscle mass	Moderate hyperinflation, widespread atelectasis and areas of infection
Severe <40	5	Orthopnea stays in chair or bed	Tachypnea, tachycardia, extensive crackles, cyanosis, severe clubbing	Marked malnutrition, rectal prolapse, etc.	Severe hyperinflation, lobar atelectasis and bronchiectasis. nodules/cysts, cardiac enlargement

This scoring system is pivotal in guiding treatment decisions and monitoring disease progression in patients with CF.

Physiotherapy and airway techniques for CF 

Physiotherapy plays a crucial role in the management of CF by enhancing airway clearance and improving respiratory function. Various airway clearance techniques (ACTs) are employed to mobilize mucus, reduce infection risk, and maintain lung function. Common techniques include postural drainage (PD), chest physiotherapy (CPT), autogenic drainage (AD), positive expiratory pressure (PEP) devices, high-frequency chest wall oscillation (HFCWO), and newer modalities like the Flutter device and intrapulmonary percussive ventilation (IPV) [50].

ACTs

Conventional CPT and techniques like PEP, HFCWO, Flutter devices, and AD effectively aid mucus clearance in CF, improving lung function and enhancing patient adherence, with varying levels of physical demand and convenience (Table 5). 

**Table 5:** Airway Clearance Techniques

Technique	Description and Effectiveness
Conventional Chest Physiotherapy (CCPT) [50]	Incorporates postural drainage, percussion, and vibration to clear mucus. Effective in improving forced expiratory volume (FEV1) and reducing mucus retention, especially during exacerbations.
Positive Expiratory Pressure (PEP) [51,52]	Uses a mask or mouthpiece to create back pressure, aiding mucus clearance and preventing airway collapse. Comparable to CCPT in improving lung function over the long term, particularly in stable patients. Its portability makes it ideal for home care.
High-Frequency Chest Wall Oscillation (HFCWO) [53]	Devices like the Vest provide external oscillations to the chest wall, facilitating mucus clearance. Comparable to CCPT in maintaining lung function while improving adherence due to user-friendly design.
Flutter Device [54,55]	Combines oscillations with PEP to assist in mucus mobilization. Regular use improves lung function and reduces hospital admissions, particularly during acute exacerbations.
Autogenic Drainage (AD) [56]	A controlled breathing technique that allows patients to modulate airflow to dislodge mucus. As effective as CCPT while being less physically demanding, which enhances patient adherence.

Effectiveness of physiotherapy vs. pharmacological interventions

While pharmacological treatments, including antibiotics and CFTR modulators, are vital in managing CF, physiotherapy remains essential for maintaining pulmonary function and preventing respiratory infections. Comparative studies indicate that ACTs can improve forced expiratory volume in one second (FEV1), forced vital capacity (FVC), and forced expiratory flow at 25-75% of the pulmonary volume (FEF25-75), particularly when combined with pharmacological therapies (Gaskin 1998; Homnick 1995) [57,58].

Impact on respiratory function and adherence

Long-term studies highlight that regular adherence to physiotherapy can significantly slow the decline in lung function, ultimately improving life expectancy for CF patients (McIlwaine 1997; Reisman 1988) [59]. Techniques that are easier for patients to perform independently, such as PEP and Flutter, tend to yield higher adherence rates. Devices facilitating self-administration also encourage better compliance, especially among pediatric patients.

Nutritional interventions

Nutritional support is critical for CF management, as approximately 85% of patients face pancreatic insufficiency. Nutritional strategies include the following (Table 6).

**Table 6:** Nutritional Intervention CF - cystic fibrosis; CFTR - cystic fibrosis transmembrane conductance regulator

Intervention	Description
Oral Calorie Supplements [60]	Used to boost daily calorie intake and support weight gain.
Managing CF-related Diabetes (CFRD) [61-63]	Insulin therapy combined with a high-calorie, high-protein, and high-fat diet is essential.
Pancreatic Enzyme Replacement Therapy [64]	Vital for normal growth in children and weight maintenance in adults; new CFTR modulators show promise for improving pancreatic function.

Management of gastrointestinal (GI) issues 

Common GI issues in CF are managed through treatments, such as ursodeoxycholic acid (UDCA) for cystic fibrosis liver disease (CFLD), probiotics to modify the microbiota profile of CF patients, and laxatives to treat constipation and influence microbiota and bile acid homeostasis [65].

Comparative analysis of treatments 

In a comparison of safety outcomes between elexacaftor/tezacaftor/ivacaftor (ELX/TEZ/IVA) (triple therapy) and tezacaftor/ivacaftor (TEZ/IVA) (dual therapy), both treatments showed similar overall incidences of adverse events (AEs), at 58% for triple therapy and 63% for dual therapy. However, only the dual therapy group reported a severe AE (2%). Serious AEs (SAEs) were also infrequent, with the triple therapy group experiencing two SAEs (4%) compared to one SAE (2%) in the dual therapy group. Cough was more common in the triple therapy group (15%) compared to dual therapy (8%), while pulmonary exacerbations were higher in the dual therapy group (12% vs. 2% for triple therapy). Elevated transaminase levels were noted only in the triple therapy group (7%), suggesting a potential safety concern not observed with dual therapy. Despite these differences, both therapies were generally well-tolerated, with no reported discontinuations due to AEs. Overall, while triple therapy may present more varied safety issues, particularly regarding elevated liver enzymes and cough frequency, both treatment options remain viable for CF management (Table 7).

**Table 7:** Safety Outcomes Comparison [66,67] AEs - adverse events; ELX/TEZ/IVA - elexacaftor/tezacaftor/ivacaftor (triple therapy); SAEs - serious adverse events; TEZ/IVA - tezacaftor/ivacaftor (dual therapy)

Safety Outcome	ELX/TEZ/IVA (Triple Therapy)	TEZ/IVA (Dual Therapy)
Adverse Events (AEs)	32 (58%)	33 (63%)
Severe AEs	0 (0%)	1 (2%)
Serious Adverse Events (SAEs)	2 (4%)	1 (2%)
Common AEs (Cough, Pulmonary Exacerbation)	Cough (15%), exacerbation (2%)	Cough (8%), exacerbation (12%)
Elevated Transaminase Levels	4 (7%)	0 (0%)
Rash	2 (4%)	2 (4%)
Discontinuation Due to AEs	0 (0%)	0 (0%)

Efficacy and safety

Monotherapy shows limited efficacy with acceptable safety, while dual therapy improves quality of life and lung function with a better safety profile. Triple therapy offers the most significant benefits and robust safety, whereas combination therapy shows similar efficacy to triple therapy but has limited safety data available (Table 8).

**Table 8:** Efficacy and Safety Profile [66,67] AEs: adverse events; BP: blood pressure; CFQ-R: Cystic Fibrosis Questionnaire-Revised; CI: confidence interval; FEV1: forced expiratory volume in one second; QoL: quality of life; VX-659: a specific drug candidate in clinical trials

Therapy Type	Efficacy	Safety Profile
Monotherapy	Limited effectiveness. No clinically relevant improvement in QoL. Slight decline in FEV1 % predicted.	Generally acceptable. Mild respiratory adverse events, particularly with lumacaftor.
Dual Therapy	Improved QoL. Meaningful increase in FEV1 % predicted. Concerns of transient breathlessness and increased BP.	Better safety profile than lumacaftor/ivacaftor. No significant increase in blood pressure.
Triple Therapy	Most substantial benefits. Remarkable increase in QoL and FEV1 % predicted. Reduced sweat chloride levels.	Robust safety profile. Mild to moderate AEs comparable to rezacaftor/ivacaftor. No significant increase in AEs.
Combination (VX-659/Tezacaftor/Ivacaftor)	Similar efficacy to triple therapy but limited data available.	Safety profile not fully characterized due to limited data.

While all therapies offer varying levels of efficacy, triple therapy (elexacaftor/tezacaftor/ivacaftor) emerges as the most effective option, showcasing superior outcomes and a strong safety profile. This indicates a significant advancement in treatment regimens for CF management

## Conclusions

In conclusion, the landscape of CF treatment has evolved significantly, driven by advances in genetic research and the development of targeted therapies. CFTR modulators, alongside innovative antibacterial approaches, represent a promising shift toward personalized medicine, addressing the underlying genetic mutations rather than merely managing symptoms. These advancements not only enhance the quality of life for patients but also hold the potential for improved long-term health outcomes, including increased life expectancy and better management of complications associated with the disorder.

As we continue to explore and validate these novel therapies through rigorous evidence-based studies, it is essential to remain vigilant in assessing their long-term efficacy and safety. By prioritizing patient-centered approaches and maintaining a focus on innovative research, we can better support individuals living with CF and improve their overall health trajectories in the face of this complex and chronic condition.

## References

[1] Brown SD, White R, Tobin P (2017). Keep them breathing: cystic fibrosis pathophysiology, diagnosis, and treatment. JAAPA.

[2] Elborn JS (2016). Cystic fibrosis. Lancet.

[3] Rowe SM, Miller S, Sorscher EJ (2005). Cystic fibrosis. N Engl J Med.

[4] Ratjen F, Bell SC, Rowe SM, Goss CH, Quittner AL, Bush A (2015). Cystic fibrosis. Nat Rev Dis Primers.

[5] (2024). Cystic Fibrosis Foundation Patient Registry 2018 Annual Data Report. Cystic Fibrosis Foundation. https://www.cff.org/sites/default/files/2021-10/2018-Annual-Report.pdf..

[6] Castellani C, Assael BM (2017). Cystic fibrosis: a clinical view. Cell Mol Life Sci.

[7] Hall JR, Rouillard KR, Suchyta DJ, Brown MD, Ahonen MJ, Schoenfisc MH (2020). Mode of nitric oxide delivery affects antibacterial action. ACS Biomater Sci Eng.

[8] Andersen DH (1938). Cystic fibrosis of the pancreas and its relation to celiac disease. A clinical and pathologic study. Am J Dis Child.

[9] Prasad SA, Tannenbaum EL, Mikelsons C (2024). Physiotherapy in cystic fibrosis. J R Soc Med.

[10] Farrell PM, Rock MJ, Baker MW (2020). The impact of the CFTR gene discovery on cystic fibrosis diagnosis, counseling, and preventive therapy. Genes (Basel).

[11] Moskowitz SM, Silva SJ, Mayer-Hamblett N (2008). Shifting patterns of inhaled antibiotic use in cystic fibrosis. Pediatr Pulmonol.

[12] VanDevanter DR, Craib ML, Pasta DJ, Millar SJ, Morgan WJ, Konstan MW (2018). Cystic fibrosis clinical characteristics associated with dornase alfa treatment regimen change. Pediatr Pulmonol.

[13] Lynch JP 3rd, Sayah DM, Belperio JA, Weigt SS (2015). Lung transplantation for cystic fibrosis: results, indications, complications, and controversies. Semin Respir Crit Care Med.

[14] Accurso FJ, Rowe SM, Clancy JP (2010). Effect of VX-770 in persons with cystic fibrosis and the G551D-CFTR mutation. N Engl J Med.

[15] Wainwright CE, Elborn JS, Ramsey BW (2015). Lumacaftor-ivacaftor in patients with cystic fibrosis homozygous for Phe508del CFTR. N Engl J Med.

[16] Jia S, Taylor-Cousar JL (2023). Cystic fibrosis modulator therapies. Annu Rev Med.

[17] Awatade NT, Wong SL, Hewson CK, Fawcett LK, Kicic A, Jaffe A, Waters SA (2018). Human primary epithelial cell models: promising tools in the era of cystic fibrosis personalized medicine. Front Pharmacol.

[18] Farinha CM, Callebaut I (2022). Molecular mechanisms of cystic fibrosis - how mutations lead to misfunction and guide therapy. Biosci Rep.

[19] Bergeron C, Cantin AM (2019). Cystic fibrosis: pathophysiology of lung disease. Semin Respir Crit Care Med.

[20] Lubamba B, Dhooghe B, Noel S, Leal T (2012). Cystic fibrosis: insight into CFTR pathophysiology and pharmacotherapy. Clin Biochem.

[21] Moskowitz SM, Chmiel JF, Sternen DL, Cheng E, Gibson RL, Marshall SG, Cutting GR (2008). Clinical practice and genetic counseling for cystic fibrosis and CFTR-related disorders. Genet Med.

[22] Ratjen F, Grasemann H (2012). New therapies in cystic fibrosis. Curr Pharm Des.

[23] Mall MA, Hartl D (2014). CFTR: cystic fibrosis and beyond. Eur Respir J.

[24] Matsui H, Grubb BR, Tarran R (1998). Evidence for periciliary liquid layer depletion, not abnormal ion composition, in the pathogenesis of cystic fibrosis airways disease. Cell.

[25] Haack A, Aragão GG, Novaes MR (2013). Pathophysiology of cystic fibrosis and drugs used in associated digestive tract diseases. World J Gastroenterol.

[26] Huang L, Lai HJ, Song J (2023). Impact of intrinsic and extrinsic risk factors on early-onset lung disease in cystic fibrosis. Pediatr Pulmonol.

[27] Sanders DB, Li Z, Laxova A (2014). Risk factors for the progression of cystic fibrosis lung disease throughout childhood. Ann Am Thorac Soc.

[28] Bell SC, Mall MA, Gutierrez H (2020). The future of cystic fibrosis care: a global perspective. Lancet Respir Med.

[29] Hale K, Brown S (2024). Pharmacologic management of cystic fibrosis. US Pharm 49.7.

[30] Lopes-Pacheco M (2019). CFTR modulators: the changing face of cystic fibrosis in the era of precision medicine. Front Pharmacol.

[31] Sermet-Gaudelus I (2013). Ivacaftor treatment in patients with cystic fibrosis and the G551D-CFTR mutation. Eur Respir Rev.

[32] (2023). Dosing and Administration for KALYDECO® (ivacaftor). http://www.kalydecohcp.com/dosing-administration.

[33] (2023). Dosing and Administration. Orkambi (lumacaftor/ivacaftor) product information. Boston, MA: Vertex Pharmaceuticals, Inc August.

[34] Lefebvre P, Lafeuille MH, Tiggelaar S (2018). Perspectives on the common drug review process at the Canadian Agency for Drugs and Technologies in Health. Decision Making in a World of Comparative Effectiveness Research.

[35] (2023). Dosing and Administration. Symdeko (tezacaftor/ivacaftor) product information. Boston, MA: Vertex Pharmaceuticals, Inc August.

[36] (2020). “Contraindications”. Symdeko, Product Monograph, Vertex Pharmaceuticals (Canada) Incorporated. https://pi.vrtx.com/files/Canadapm_symdeko_en.pdf.

[37] Ridley K, Condren M (2020). Elexacaftor-tezacaftor-ivacaftor: the first triple-combination cystic fibrosis transmembrane conductance regulator modulating therapy. J Pediatr Pharmacol Ther.

[38] (2024). Dosage and Administration. Highlights of prescribing information. https://www.accessdata.fda.gov/drugsatfda_docs/label/2021/212273s004lbl.pdf.

[39] (2024). Dosage and Administration. Highlights of prescribing information, Pulmozyme (Dornase alpha) product information. South San Francisco, CA: Genentech, Inc February.

[40] Wark P, McDonald VM (2018). Nebulised hypertonic saline for cystic fibrosis. Cochrane Database Syst Rev.

[41] Gupta HV, Gupta VV, Kaur G (2016). Effectiveness of 3% hypertonic saline nebulization in acute bronchiolitis among Indian children: a quasi-experimental study. Perspect Clin Res.

[42] Retsch-Bogart GZ, Quittner AL, Gibson RL, Oermann CM, McCoy KS, Montgomery AB, Cooper PJ (2009). Efficacy and safety of inhaled aztreonam lysine for airway pseudomonas in cystic fibrosis. Chest.

[43] Cigana C, Nicolis E, Pasetto M, Assael BM, Melotti P (2006). Anti-inflammatory effects of azithromycin in cystic fibrosis airway epithelial cells. Biochem Biophys Res Commun.

[44] (2024). Dosage and Administration. Highlights of prescribing information, Tobi Podhaler (tobramycin inhalation powder) product information. San Carlos, CA: Mylan Pharmaceuticals, Inc February.

[45] (2024). Dosage and Administration. Highlights of prescribing information, Tobi (tobramycin) product information. East Hanover, NJ: Novartis Pharmaceuticals, Inc February.

[46] (2019). Dosage and Administration. Highlights of prescribing information, Cayston (aztreonam for inhalation) product information. https://www.gilead.com/-/media/files/pdfs/medicines/respiratory/cayston/cayston_pi.pdf.

[47] Meyer M, Huaux F, Gavilanes X (2009). Azithromycin reduces exaggerated cytokine production by M1 alveolar macrophages in cystic fibrosis. Am J Respir Cell Mol Biol.

[48] Hafen GM, Ranganathan SC, Robertson CF, Robinson PJ (2006). Clinical scoring systems in cystic fibrosis. Pediatr Pulmonol.

[49] Khalilzadeh S (2013). Shwachman score in clinical evaluation of cystic fibrosis. J Compr Ped.

[50] Arens R, Gozal D, Omlin KJ, Vega J, Boyd KP, Keens TG, Woo MS (1994). Comparison of high frequency chest compression and conventional chest physiotherapy in hospitalized patients with cystic fibrosis. Am J Respir Crit Care Med.

[51] McIlwaine M, Button B, Dwan K (2015). Positive expiratory pressure physiotherapy for airway clearance in people with cystic fibrosis. Cochrane Database Syst Rev.

[52] Rocamora-Pérez P, Benzo-Iglesias MJ, Valverde-Martínez MLÁ, García-Luengo AV, Aguilar-Parra JM, Trigueros R, López-Liria R (2022). Effectiveness of positive expiratory pressure on patients over 16 years of age with cystic fibrosis: systematic review and meta-analysis. Ther Adv Respir Dis.

[53] Bradley JM (2010). High frequency chest wall oscillation in cystic fibrosis. Thorax.

[54] Gondor M (1999). Comparison of flutter device and chest physical therapy in the treatment of cystic fibrosis pulmonary exacerbation. Pediatr Pulmonol.

[55] Homnick DN (1998). Comparison of Flutter device and chest physiotherapy in the treatment of cystic fibrosis pulmonary exacerbation. Pediatr Pulmonol.

[56] McIlwaine M (2010). Long-term comparative trial of conventional postural drainage and autogenic drainage in cystic fibrosis. Eur Respir J.

[57] Thee S, Stahl M, Fischer R (2021). A multi-centre, randomized, controlled trial on coaching and telemonitoring in patients with cystic fibrosis: conneCT CF. BMC Pulm Med.

[58] van Winden CM, Visser A, Hop W, Sterk PJ, Beckers S, de Jongste JC (1998). Effects of flutter and PEP mask physiotherapy on symptoms and lung function in children with cystic fibrosis. Eur Respir J.

[59] Reisman JJ (1988). Role of physiotherapy in cystic fibrosis: a 5-year prospective study. J Pediatr.

[60] Smyth R, Walters S (2000). Oral calorie supplements for cystic fibrosis. Cochrane Database Syst Rev.

[61] Kaminski BA, Goldsweig BK, Sidhaye A, Blackman SM, Schindler T, Moran A (2019). Cystic fibrosis related diabetes: nutrition and growth considerations. J Cyst Fibros.

[62] Alves C, Della-Manna T, Albuquerque CT (2020). Cystic fibrosis-related diabetes: an update on pathophysiology, diagnosis, and treatment. J Pediatr Endocrinol Metab.

[63] Lurquin F, Buysschaert M, Preumont V (2023). Advances in cystic fibrosis-related diabetes: current status and future directions. Diabetes Metab Syndr.

[64] Singh VK, Schwarzenberg SJ (2017). Pancreatic insufficiency in cystic fibrosis. J Cyst Fibros.

[65] van de Peppel IP, Bodewes FA, Verkade HJ, Jonker JW (2019). Bile acid homeostasis in gastrointestinal and metabolic complications of cystic fibrosis. J Cyst Fibros.

[66] Heijerman HG, McKone EF, Downey DG (2019). Efficacy and safety of the elexacaftor plus tezacaftor plus ivacaftor combination regimen in people with cystic fibrosis homozygous for the F508del mutation: a double-blind, randomised, phase 3 trial. Lancet.

[67] Southern KW, Murphy J, Sinha IP, Nevitt SJ (2020). Corrector therapies (with or without potentiators) for people with cystic fibrosis with class II CFTR gene variants (most commonly F508del). Cochrane Database Syst Rev.

